# The Midline Protein Regulates Axon Guidance by Blocking the Reiteration of Neuroblast Rows within the Drosophila Ventral Nerve Cord

**DOI:** 10.1371/journal.pgen.1004050

**Published:** 2013-12-26

**Authors:** Mary Ann Manavalan, Ivana Gaziova, Krishna Moorthi Bhat

**Affiliations:** Department of Neuroscience and Cell Biology, University of Texas Medical Branch School of Medicine, Galveston, Texas, United States of America; New York University, United States of America

## Abstract

Guiding axon growth cones towards their targets is a fundamental process that occurs in a developing nervous system. Several major signaling systems are involved in axon-guidance, and disruption of these systems causes axon-guidance defects. However, the specific role of the environment in which axons navigate in regulating axon-guidance has not been examined in detail. In Drosophila, the ventral nerve cord is divided into segments, and half-segments and the precursor neuroblasts are formed in rows and columns in individual half-segments. The row-wise expression of segment-polarity genes within the neuroectoderm provides the initial row-wise identity to neuroblasts. Here, we show that in embryos mutant for the gene *midline*, which encodes a T-box DNA binding protein, row-2 neuroblasts and their neuroectoderm adopt a row-5 identity. This reiteration of row-5 ultimately creates a non-permissive zone or a barrier, which prevents the extension of interneuronal longitudinal tracts along their normal anterior-posterior path. While we do not know the nature of the barrier, the axon tracts either stall when they reach this region or project across the midline or towards the periphery along this zone. Previously, we had shown that *midline* ensures ancestry-dependent fate specification in a neuronal lineage. These results provide the molecular basis for the axon guidance defects in *midline* mutants and the significance of proper specification of the environment to axon-guidance. These results also reveal the importance of segmental polarity in guiding axons from one segment to the next, and a link between establishment of broad segmental identity and axon guidance.

## Author Summary

During nervous system development, once formed from neuroblasts, neurons grow axons in the direction of their synaptic partners. Genetic forces guide these axon growth cones towards the target. This is known as axon guidance or pathfinding. There are a number of proteins that regulate axon-pathfinding. The well-known examples are the Slit and its receptor Roundabout, and Netrin and its receptor Frazzled. The Drosophila embryo and the nervous system are divided into segments by segmentation genes. Mutations in segmentation genes affect axon guidance, although how they do so is not well understood. In our work described here, we show that the T-box protein Midline prevents mis-specification of neuroblast rows, in particular, it prevents row 2 from becoming row 5. Thus, in *midline* mutants, row 2 changes into row 5, ultimately creating a non-permissive barrier that prevents axons from following their defined path. Thus, axons stop and diverge when they reach this barrier. Our results show how mutations in segmentation genes can affect axon guidance and how significant the environment is for axon-pathfinding. Our work is also a cautionary reminder that guidance defects need to be interpreted with care and can arise due to a variety of other defects.

## Introduction

In the Drosophila nerve cord, about 20 longitudinal axon tracts on either side of the midline, each consisting of axons from several neurons, connect different segments with one another. Several direct players in axon guidance have been identified. For example, previous studies have shown that mutations in two signaling pathways, the ligand Slit (Sli) and its receptors Roundabouts (Robo, Robo2 and Robo3) and the ligand Netrin (Net) and its receptor Frazzled (Fra; the vertebrate homologue is known as Deleted in Colorectal cancers or DCC) disrupt the precise positioning of these tracts by altering their growth cone guidance [1]–[7]. Whenever the Slit system is disrupted, longitudinal axon tracts inappropriately cross the midline [1], whereas with the disruption of the Net-Fra system, which primarily mediates the attraction of commissural tracts to facilitate their midline crossing [4]–[6], a large number of commissural growth cones fail to cross the midline [4], [5], [8].

There is a second set of players not linked to the direct players such as Slit-Robo or Net-Fra, but cause axon guidance defects when disrupted. In these mutants, the pioneering axon growth cones fail, either due to the absence of the neurons themselves or due to a mis-specification of their identity. As a result, follower neurons fail to properly project their growth cones along the correct trajectories. For instance, when the pioneering neurons pCC or vMP2 are either ablated [9] or mis-specified [10], the follower axon tracts cross the midline, ignoring the guidance cues mediated by Slit and Robo [10].

It is obvious that the environment in which growth cones travel would have an impact on axon guidance. However, it is not clear in what specific way the environment in which axons travel influence axon guidance or how specific the influence would be on axon guidance. The environment is defined by cells, which express guidance determinants on their surface or release cues into the extracellular matrix. Segmentation genes, in particular segment polarity genes, broadly define the environment in which axons travel by specifying cellular identity, which then by expressing specific genes regulate guidance of specific growth cones. Segment polarity genes are expressed in rows and columns within the nerve cord and mutational analysis indicates that they specify the initial NB identity along the rows and columns [11]–[15]. For instance, row 5 identity is set mainly by the expression of Wg and Gsb (all row 5 cells express these genes), whereas row 4 is determined by the expression of Patched (Ptc) in row 4, Wg in row 5, and the absence of expression of Gsb in row 4 [reviewed in ref. 15]. Loss of function for these genes alters the identity of NBs along the entire rows. Thus, loss of function for Ptc changes row 4 into row 5, loss of Gsb changes row 5 into row 4, and loss of Wg alters rows 5, 6 and 4 identities (non-cell autonomous function of Wg also confers row identity to adjacent rows) [11]–[15]. Their expression persists in successive divisions of NBs, even as NB-specific expression of transcription factors changes following each division of a NB [16], [17].

Loss of function for these genes also cause axon guidance defects [18]. However, we do not know if the axon guidance defects in segmentation mutants are due to mis-specification of a pioneering neuronal identity, or broad changes in the environment in which axons travel (or both). Given that growth cones interact with the environmental niche along their path, it is reasonable to suppose that broad changes in the local environment can affect axon pathways. However, separating neuronal identity from changes in the environment in influencing axon guidance has been experimentally difficult.

We have been studying a gene called *midline* (*mid*), which belongs to a class of transcription factors known as T-box binding (Tbx) proteins. Tbx proteins are highly conserved among metazoans and are defined by the presence of a T-box domain, a 180–230 amino acid DNA-binding domain. Tbx proteins bind to a T-Box element (TBE), a 20-bp degenerate palindromic sequence [19]. However, TBEs are highly variable in sequence, number and distribution within promoters and Tbx proteins diverge significantly in their sequence preference [20]. Tbx proteins are known to repress transcription [21]. Moreover, mutations in *Tbx* genes can be haploinsufficient, i.e. developmental processes are sensitive to the levels of some Tbx proteins. For example, upper limb malformation and congenital heart defects in Holt-Oram syndrome are due to haploinsufficiency for TBX5 [22], [23]. Haploinsufficiency for mouse brachyury and human TBX3 and TBX1 genes causes dominant phenotypes such as short tails/tailless, Ulnar-Mammary syndrome and DiGeorge syndrome, respectively [23], [24].

In *Drosophila*, loss of function for *mid* (also known as *lost in space* or *los*, or *extra*) was initially shown to cause cuticle defects in the midline region of the embryo, thus the name *midline* [25]. Subsequently, it has been shown that *mid* mutants also cause heart defects [26], defects in the lateral chordotonal axons, and shorter and defasciculated dorsally routed axons in the peripheral nervous system (PNS) [5]. We recently showed that Mid ensures ancestry-dependent fate specification of a GMC, i.e, fate of a GMC is changed without affecting the parent NB identity, thus overriding the GMC's ancestry [20]. Thus, in *mid* mutants, a GMC from an unrelated NB (we have named it the M-lineage, M for Mid) changes into GMC-1 (also known as GMC4-2a) of the RP2/sib lineage without altering the parent NB identity. Also, this occurs several hours after the window of time in which the bona fide GMC-1 of the RP2/sib cells is formed.

A subsequent study by Liu et al. [27] reported that ectopic expression of *mid* in salivary gland can ectopically induce expression of *robo, slit, Netrin and frazzled*. The implication is that Mid regulates axon guidance via regulation of these guidance genes and that the axon guidance defects observed in *mid* loss of function mutants are due to loss of expression of these guidance genes. However, the regulation of these genes by Mid in salivary glands, where none of these axon guidance genes including *mid* are normally expressed, is of no functional significance. Mid must regulate these genes in the nerve cord to be of relevance. Moreover, the mostly non-overlapping expression patterns of *mid, robo* and *slit* in the developing CNS, save a few cells in the lateral region of the nerve cord as reported by Liu et al [27] does not make sense with a functional direct transcriptional regulatory role for Mid on these genes during axon guidance.

We sought to explore these issues, including the possibility of an indirect regulation of axon guidance genes by *mid*, with the aim to understand the molecular basis for the guidance defects in *mid* mutants. We show here that the primary axon guidance defect in *mid* mutant embryos is stalling of axon tracts midway between the posterior commissure (PC) and the anterior commissure (AC) of the next segment, with tracts often crossing the midline, or projecting peripherally outward, perpendicular to the midline. This defect is due to the transformation of row 2 NBs and their precursor neuroectodermal (NE) cells, which are located midway between the PC and the AC of the next segment, into row 5 cells. Row 5 is normally located at the level of the PC and defines the parasegmental boundary (PSB). The fact that axon tracts stall or project across the midline or towards the periphery precisely along this transformed row, indicates that these newly re-specified row 5 cells creates an unsuitable or inhibitory niche for these pioneering axons to navigate along the midline. These results argue that the role of Mid in regulating axon guidance is indirect and via proper specification of row identity within the nerve cord. Our results also show that Mid does not regulate transcription of *frazzled, sli* or *robo*, directly or indirectly, in cells where their expression matters. These results provide novel insight into how segmentation or row identity facilitate axon guidance later in neurogenesis and distinguishes how broad environmental identities, as opposed to individual neuronal identity, influence axon guidance.

## Results

### Temporal axon guidance defects in *mid* mutants differ from those in *slit* and *robo* mutants

**Figure 1:**
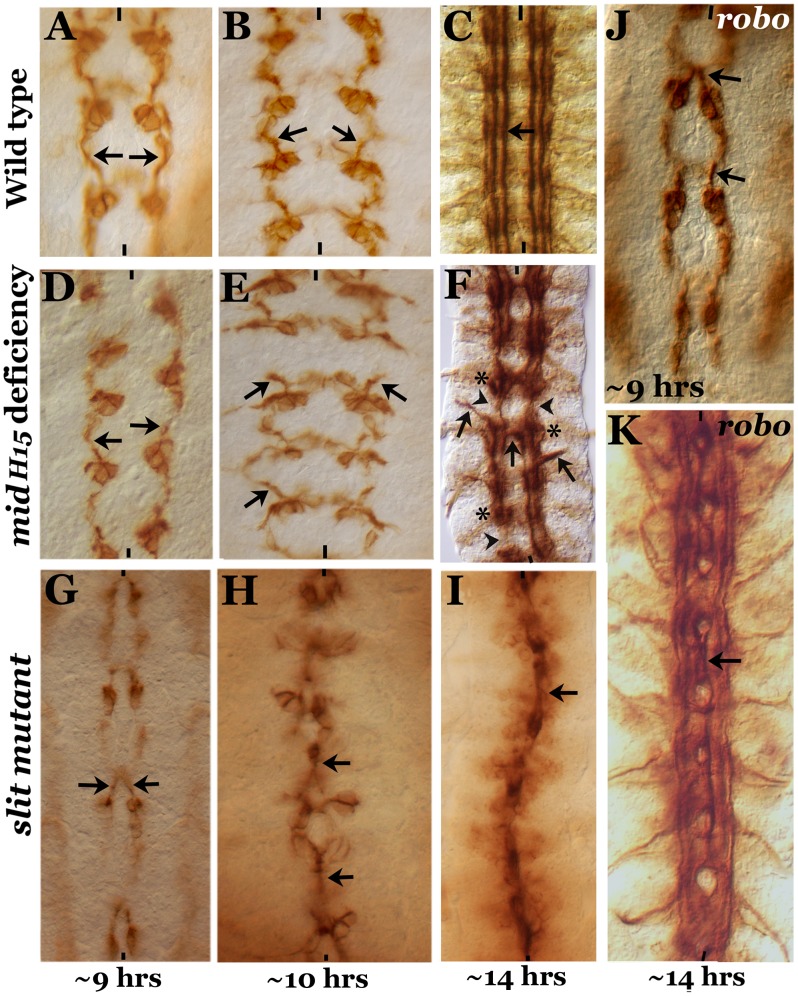
Comparison of axon guidance defects in *mid*, *slit* and *robo* mutants. Embryos from wild type (panels A–C), *mid H15* deficiency (panels D–F), *slit* (panels G–I) and *robo* (panels J, K) were stained with an antibody against Fas II. Arrow indicates projection from the pCC neuron as well as the medial tract (in older embryos) or its abnormal crossing at the midline; arrowheads in panel F indicate breaks, and star indicates groups of stalled longitudinal axon tracts. These *mid*-specific defects are not seen in either *slit* or *robo* mutant embryos. Anterior end is up, midline is marked by vertical lines. Note that to define age of the embryo we prefer “hours” of development (at room temperature) to the traditional “stages” of development. We think that this is particularly important for describing events at the molecular level. Assigning different “stages” was done based on gross morphological milestones during embryogenesis, and each stage can be 10 minutes short or 2–3 hours long. For defining age or development at the molecular level this approach is not meaningful and can be misleading.

Previous results have indicated that embryos mutant for *mid* show axon guidance defects [5]. We sought to examine in detail the axon guidance defects in *mid* mutants in the embryonic CNS during development and compare those defects to the defects at corresponding developmental stages in *slit* and *robo* mutants. As shown in Fig. 1, embryos of different developmental stages were stained for Fasciclin II (Fas II) positive axon pathways using an antibody against Fas II. Fas II staining of ∼9 hours old embryos reveals the nascent medial tract, which is closest to the midline and is pioneered by the growth cone from pCC (arrows in Fig. 1A, wild type). In ∼9 hours old *mid* deficiency embryos the pCC growth cones were the same as in wild type, projecting slightly outward and then parallel to the midline (Fig. 1D, arrows). However, in ∼9 hours old *slit* mutant embryos, the pCC growth cones projected directly towards the midline (arrows in Fig. 1G). In ∼9 hours old *robo* mutant embryos, the pCC growth cones also projected towards the midline, although the defects were less severe than in *slit* embryos (Fig. 1J, arrows).

By 10 hours of development, the growth cones from pCC in *mid* embryos were projecting outward and away from the midline as if they had encountered an inhibitory zone (Fig. 1E, arrows, compare with wild type, Fig. 1B), whereas in ∼10 hours old *slit* mutant embryos, the growth cones from pCC were fasciculated with each other at the midline (Fig. 1H). By ∼14 hours of development in *mid* embryos, the three different Fas II tracts, the medial tract, the intermediate tract and the lateral tract, all run parallel to the midline, could be seen with similar spacing between each other and from the midline, as in wild type (Fig. 1F to 1C). However, as shown in Fig. 1F, in ∼14 hours old *mid* mutant embryos we could observe tracts inappropriately projecting outward (thick arrow), breaks or missing tracts along the longitudinal axis (arrowhead) and crossing the midline (midline arrow). We could also observe stalled growth cones forming a blob of axon tracts along the nerve cord (Fig. 1C, star, see also Table 1).

**Table 1:** Penetrance of the axon tracts defects in *mid* mutants.

Genotype	Hemisegments with axon guidance defects (%)			No. of hemisegments counted
	Stalling	Midline crossing	Peripheral projection	
*Wild type*	0	1	0	280
*mid^1^*	73	*29*	*31*	240
*los^1^*	75	27	27	240
*H15, mid^df^*	83	36	25	240
*slit*	0	100	5	240^*^
*robo*	0	79	0	240^*^
*^#^mid/+;+/sli*	0	0	0	750
*^#^fra/+;+/los1*	0	0	0	923
*^#^Fra/+;+/H15, mid^df^*	0	0	0	637
*^#^mid/+;+/sli*	0	0	0	750
*^#^fra/+;+/los1*	0	0	0	923
*^#^Fra/+;+/H15, mid^df^*	0	0	0	637
*mid/+;+/sli*	0	0	0	750
*fra/+;+/los1*	0	0	0	923
*Fra/+;+/H15, mid^df^*	0	0	0	637

Embryos were stained with BP102 (for stalling) and Fas II (for midline crossing and peripheral projection) and examined for axon guidance defects. Star (*) indicates approximation of number of hemisegments counted since it is impossible to count the hemisegments accurately in these mutants (*sli* and *robo*) due to the collapsing of tracts at the midline. Number sign (#) indicates parents without any balancers (mutant flies were crossed to wild type and the progeny males and females carrying the mutant chromosome over wild type chromosome from this cross were inter-crossed to generate homozygous mutant embryos). The remaining genotypes were all obtained from balancer-bearing flies. Note that a given hemisegment can have all the three scored phenotypes.

While in ∼14 hours old *slit* mutant embryos the three tracts were all collapsed at the midline (Fig. 1I), in *robo* mutants, the medial tract was mostly fused at the midline, with the other two tracts more or less normal (Fig. 1K)(the partial penetrance of the guidance defects is due to redundancy with Robo2 and Robo3 receptors) [2], [3], . The frequency of various guidance defects in *mid, slit* and *robo* mutants are presented in Table 1. These results indicate that axon guidance defects in *mid* mutants are significantly different from axon guidance defects in s*lit* and *robo* mutants. If Mid regulates axon guidance via regulating *slit* and *robo*, the guidance phenotypes in all the three mutants should fall more or less into the same general category. Our above results show that this is not the case and argues against the possibility that Mid regulates *slit* and *robo* and that the axon guidance defects in *mid* mutants are due loss of function for these axon guidance genes.

### Inter-neuronal axon pathways but not motoneuronal pathways are aberrant in *mid* mutants

**Figure 2:**
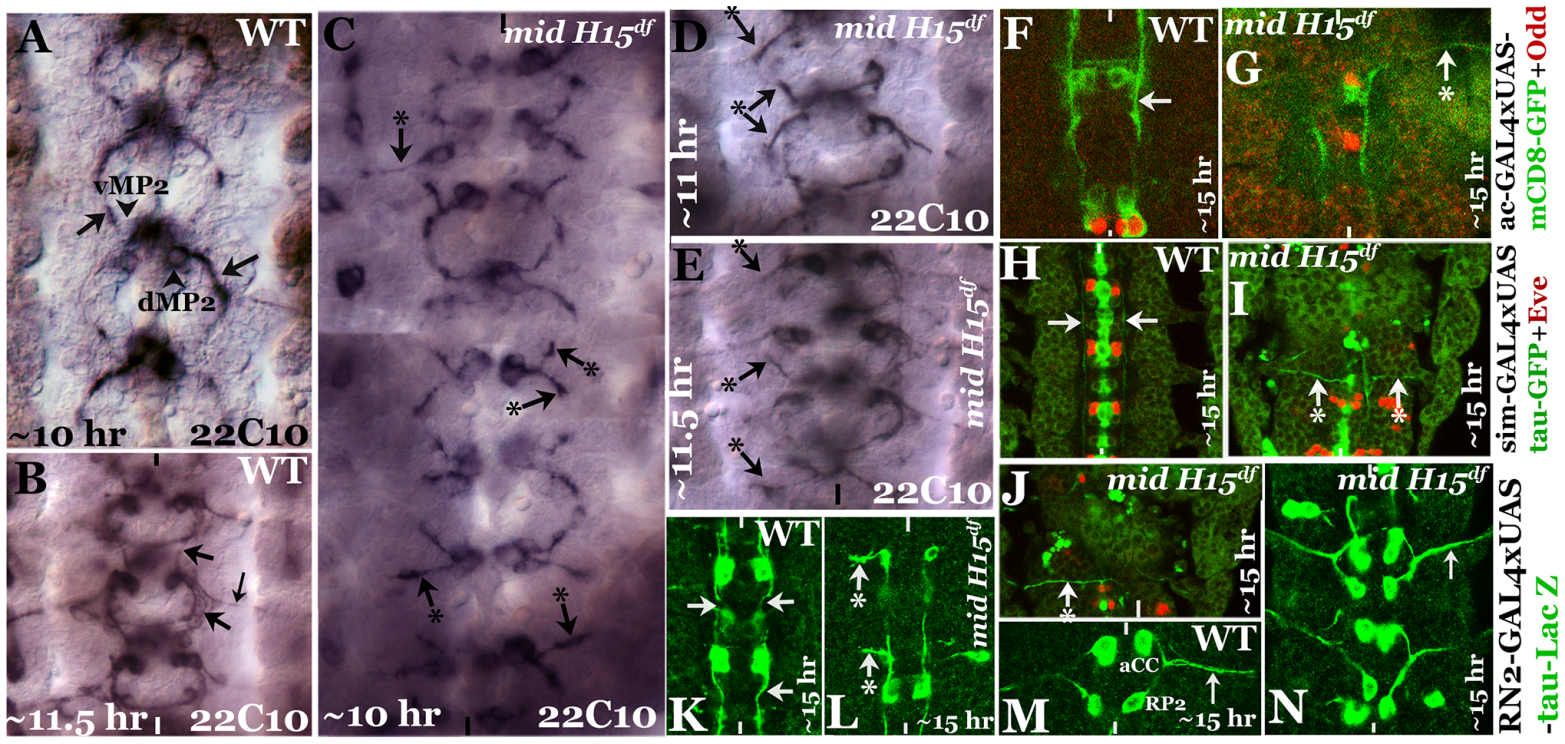
Patterns of aberrant axon projections in *mid* mutant embryos. Anterior end is up, the midline is marked by vertical lines. Embryos in **panels A–E** were stained with Mab 22C10 to visualize vMP2 and dMP2 neurons and their projections. Arrow indicates a normal projection in wild type (A, B), arrow with star indicates an abnormal/stalled projection in the mutant from dMP2 and vMP2 neurons (panels C–E). **Panel F, G**: Embryos were double-stained for GFP (green) and Odd-skipped (Odd, red). Arrow indicates normal projection from vMP2 in wild type (F), arrow with star indicates projection from vMP2 abnormally exiting the nerve cord in the mutant (G). **Panels H, I, J:** Embryos were double-stained for GFP (green) and Eve (red). Arrow indicates normal projections from MP1 in wild type (H), arrow with star indicates abnormal MP1 projection in the mutant (I, J). **Panels K, L:** Embryos were stained for LacZ (green). Arrows indicate normal projections from pCC in wild type (K), arrow with star indicates abnormal projection from pCC in the mutant (L). **Panels M, N:** Embryos were stained for LacZ (green). Smaller arrow indicates normal motor intersegmental nerve bundle from aCC and RP2 neurons in wild type (M) and in the mutant (N).

We next sought to determine the growth cone projections from vMP2, dMP2, MP1, pCC and aCC neurons in *mid* mutant embryos using more selective markers. We chose to examine the growth cones from these neurons since these neurons send out pioneering growth cones. For example, the anteriorly projecting growth cones from vMP2 and pCC pioneer the medial Fas II tract to meet the homologous axons from the next anterior segment [9], [10]. Similarly, the posteriorly projecting growth cones from MP1 and dMP2 pioneer the lateral Fas II tract to meet up with the homologous axons from the next posterior segment. Therefore, first we stained mutant embryos with a monoclonal antibody 22C10, which is raised against MAPIB. In a ∼10 hours old embryo, vMP2 (Fig. 2A) projects its growth cone anteriorly (arrow), while dMP2 projects posteriorly (arrow)(Fig. 2A). By ∼11.5 hours of development, 22C10 antibody staining revealed a fasciculated, more mature medial tract (Fig. 2B, upper arrow) and lateral tract (lower arrow), as well as several other axon pathways including the motor pathway of the aCC and RP2 neurons, both fasciculated together to form the intersegmental nerve bundle before exiting the CNS (smaller arrow).

In *mid* mutant embryos, both vMP2 and dMP2 neurons are normally formed, but we observed two key defects in their projection pattern: the growth cones often projected away in a posterior-lateral pathway similar to and/or sometimes part of the aCC-pioneered intersegmental nerve bundle (Fig. 2C, top, left arrow with star). The projections were either stalled or projected away like a motor pathway (Fig. 2C, D, E, arrow and arrow with a star). These aberrant projection patterns suggest that these growth cones have come upon a non-permissive barrier or a zone of repulsion and cannot travel in their normal path. They either stall and or project away. We further examined the projection pattern from vMP2 by expressing mCD8-GFP (mCD8 targets GFP to membrane) using the achaete (ac)-GAL4 driver. While in the wild type the axon tract from vMP2 is projected along the midline (Fig. 2F, arrow), in the mutant the projection is diverted away and perpendicular to the midline in a pathway towards the periphery, often exiting the nerve cord (Fig. 2G, arrow with star).

We next examined the projection from MP1 by expressing tau-GFP (tau directs GFP to microtubules) using the sim-GAL4 driver. While in the wild type the axon tract from MP1 is projected along the midline (Fig. 2H arrow), in the mutant the projection is diverted towards the periphery, perpendicular to the midline (Fig. 2I, J, arrow with star). This aberrant projection defect in MP1 was highly penetrant and severe.

We next examined the growth cone projection from pCC by expressing UAS-tau-lacZ transgene in pCC neuron using the RN2-GAL4 driver. This driver drives the tau-lacZ in pCC neuron (Fig. 2K, L; it also drives in aCC and RP2, Fig. 2M, N). As shown in Fig. 2K, in the wild type the pCC projects its axon anteriorly along the midline (arrow). However, in the mutant, the projection is diverted away towards the periphery perpendicular to the midline (Fig. 2L, arrow with star). We also examined the two motor pathways from neurons aCC and RP2, but did not observe any defects in their pathfinding (Fig. 2N). These results indicate that the defects are mostly confined to axon tracts from interneurons. These defects are unlikely due to a negative effect on axon growth, instead, the projections appear to encounter a barrier in their normal path and travel in an aberrant path as defined by this barrier.

### A marginal effect on the levels of Slit protein but not *slit* transcription in *mid* mutant embryos

**Figure 3:**
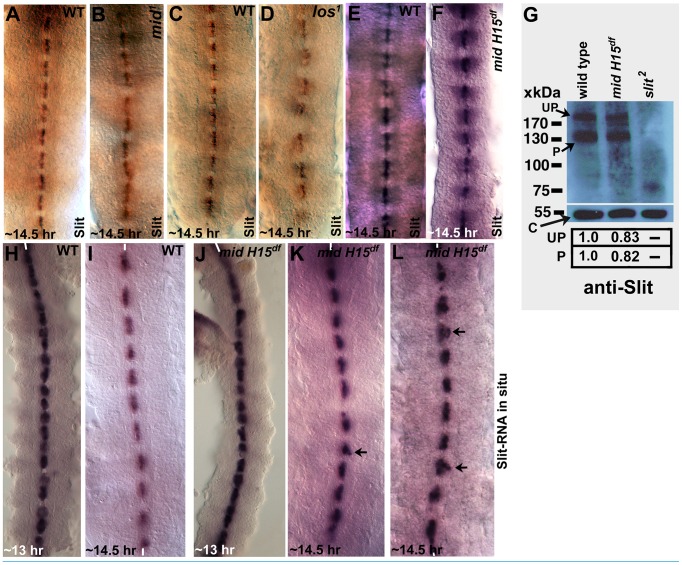
Expression of Slit protein and *slit* transcription in different *mid* mutants. Embryos from wild type (panels A, C and E), two *mid* mutant alleles (*mid^1^* and *los^1^; panels B and D*) and *mid H15* deficiency (*panel F*) were stained with an antibody against Slit. In Panel G, Western blotting analysis of Slit expression in wild type, *mid H15* deficiency and *sli^2^* mutant embryos is shown using an antibody raised against the N-terminal portion of Slit [see ref. 7]. The levels of the unprocessed (UP) and processed (P) N-terminal fragment of Slit were quantified using the AlphaEase FC software. Levels of the β-Tubulin (∼55 kDa band), determined by probing the same blot with an antibody against β-Tubulin, was used as a loading control. In panels H–L, transcription of *slit* was examined in wild type (panels H and I) and in *mid H15* deficiency embryos with whole mount RNA in situ using a probe against *slit*. Anterior end is up, midline is marked by vertical lines. Arrows in panels K and L indicate occasional disorganization or displacement of the *slit*-expressing midline glial cells in *mid* mutants.

The above results show that the axon guidance defects in *mid* are fundamentally different from those in *slit* or *robo* mutant embryos. However, given the recent report that Mid ectopically regulates *sli* and *robo* transcription in salivary glands [27], we sought to examine *mid* mutant embryos for the expression of these genes in cells where they are normally expressed. If one of the functions of Mid in wild type is to regulate expression of *slit* and *robo* genes, a significant reduction in the levels of Sli and Robo proteins should be observed in their respective domains in loss of function *mid* mutant embryos. First, we stained *mid* mutant embryos with a Slit antibody. As shown in Fig. 3 (A, C and E) in wild type, Slit is present at high levels in midline glial cells where the axon tracts of AC and PC cross the midline. It is also present in commissural and longitudinal tracts due to movement of Slit from the midline to the axon tracts via the commissural tracts [7]. We examined the two alleles of *mid* (*mid^1^* and *los^1^*)(Fig. 3B and D) and the *mid H15* deficiency (which removes both *mid* and its sister gene *H15*)(Fig. 3F). We have reported previously [20] that the *mid^1^* allele has a stop codon at amino acid (aa) position 128 (this allele is likely the strongest loss of function *mid* mutant allele) and *los^1^* has a 22 base-pair deletion resulting in a deletion of 7 aa at position 321, as well as a frame shift leading to a stop codon at aa 350 (thus, in addition to the truncation the mutant protein in this allele has 28 amino acids that are entirely different from the wild type gene; this has the potential to cause gain of function/neomorphic effects in addition to loss of function effects).

The Slit protein level was not significantly affected in homozygous *mid^1^* allele nor was it affected in the homozygous *mid H15* deficiency embryos (Fig. 3B and 3F); a marginal reduction in the levels of Slit protein was observed in *los^1^* embryos in the PC region (Fig. 3D). Whether this is due to a *los^1^*-specific gain of function effect or a background effect is not known. We further examined if the levels of the Slit protein is affected in younger stage embryos from *mid^1^*, *los^1^*, and the *mid H15* deficiency. However, no reduction in the levels of the Slit protein was observed in these alleles (data not shown). To quantify the level of Slit between wild type and the mutant embryos, we performed Western analysis of Slit in the *mid H15* deficiency embryos. The results reproducibly showed only a marginal reduction in the amount of Slit (Fig. 3G). One possibility for this slight reduction in Slit protein levels is that Liu et al [27] had reported that there is an overlapping expression of Mid and Slit in a few neurons located laterally within the nerve cord. It is possible that Mid regulates *slit* expression in these cells and that the slight reduction on Westerns reflect this regulation. Alternatively, the slight reduction in the levels as seen in Western blots is due to indirect effect of loss of function for *mid* and *H15* genes, such as mis-specification of relevant neurons/glia.

Since Mid is a transcription factor, we next sought to determine if the transcription of the *slit* gene is affected in *mid* mutant embryos by performing whole mount *slit* RNA in situ. If Mid regulates *slit* transcription at least in the PC region, where *mid* is expressed, we should observe loss of *slit* transcription in these midline cells in *mid* mutant embryos. However, as shown in Fig. 3J, K and L), no such effect on the transcription of the *slit* gene by loss of function for *mid* was observed.

### Expression of Robo is not regulated by Mid in the CNS

**Figure 4:**
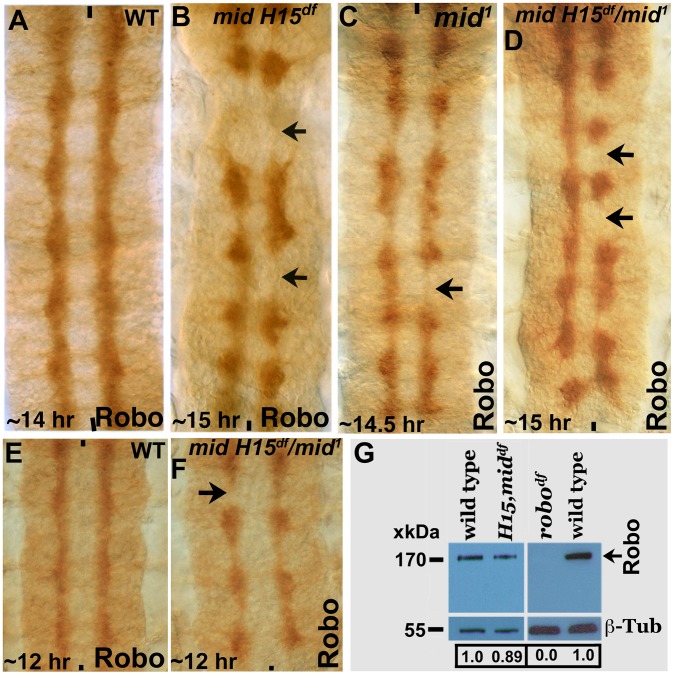
Expression of Robo is not affected in *mid* mutant embryos. Wild type (panels A and E) and *mid H15* deficiency (panel B), *mid^1^* (panel C), *mid H15^df^/mid^1^* transheterozygous (panels D and F) embryos were stained with an antibody against Robo. Anterior end is up, midline is marked by vertical lines. Severe axon tract disruptions/breaks were observed in mutant embryos but the Robo levels were similar to wild type. In panel G, Western blotting analysis of Robo in wild type, *mid H15* deficiency and *robo* deficiency embryos is shown. Note that the Robo protein band is not seen in *robo*-deficiency embryos indicating that the antibody is specific to Robo. β-Tubulin was used as a loading control and the levels were quantified using the AlphaEase FC software.

We next examined the expression of Robo in *mid^1^*, *mid H15* deficiency, and in embryos transheterozygous for the *mid H15* deficiency and *mid^1^* alleles using an antibody against Robo (Robo levels were also examined in *los^1^* allele, see later section). As shown in Fig. 4A, in wild type Robo is expressed in longitudinal pathways and is also present very weakly in AC and PC (due to incomplete down-regulation of Robo by a Commissureless protein-mediated process in commissural tracts [1]). In *mid* mutant embryonic CNS, the levels of Robo was not affected in any significant way (Fig. 4B); the lack of Robo staining in tracts (arrows, Fig. 4B) is due to the absence of axon tracts themselves. We also examined the expression of Robo in *mid H15* deficiency embryos by Western analysis, which indicated a slight reduction in the levels of the Robo protein relative to wild type (Fig. 4G). This reduction is likely due to a secondary effect originating from the breaks in axon tracts or loss of Robo-expressing cells [due to identity changes, see ref. 20] as opposed to a direct Mid regulation of *robo*.

**Figure 5:**
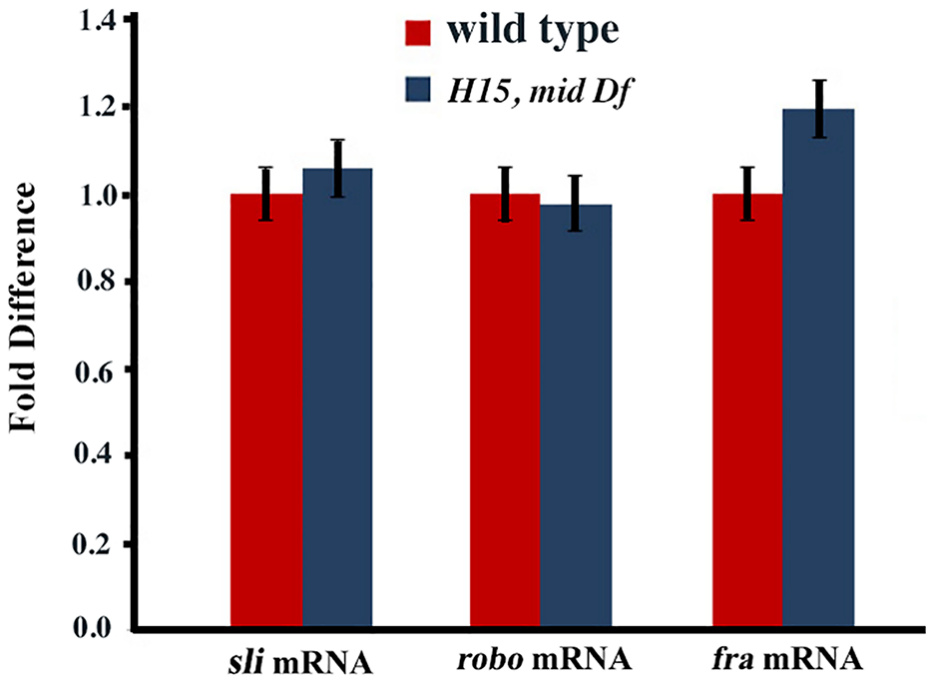
Real time quantitative pCR for *slit*, *robo* and *frazzled*. The qRT-PCR was done for samples from three seperate embryo collections for each genotype and in triplicates for each collection-sample. Standard errors (SE) were calculated from the pooled data for each genotype.

We also examined the transcription of *sli*, *robo* and *frazzled (fra)* in *mid H15* deficiency embryos using the sensitive qRT-PCR method. As shown in Fig. 5, no significant differences were detected in the transcription of any of these genes in *mid H15* mutant embryos compared to wild type. These qPCR results were reproducible using three different samples of embryo RNA preparations prepared separately in three different days, and qPCR was done in triplicates for each of the samples (the averages with standard errors were shown in Fig. 5). These results suggest that Mid has no role in the transcription of these genes during neurogenesis (note that there is no maternal contribution of *mid*).

Finally, Liu et al [27] had suggested that *mid* and *fra* show transheterozygous genetic interaction since they found that embryos transheterozygous for *mid* and *fra* have strong axon guidance defects. We re-examined if the two mutations show such an interaction by staining *mid/+, fra/+* embryos from *mid/CyO* and *fra/CyO* with Fas II and BP102 antibodies. However, we did not observe any axon guidance defects in these embryos. Sometimes, balancer-bearing parents generate a few embryos that show axon guidance (or other) defects. We have previously named this ‘balancer-induced parental effect’ [7], [20]. This effect can also be suppressive. Therefore, we generated transheterozygous embryos from *mid* and *fra* parents that do not carry any balancers (*mid/+* and *fra/+*). The transheterozygous embryos from this cross also did not have any axon guidance defects (Table 1). We did not find any axon guidance defects in embryos transheterozygous for the *mid H15* deficiency and *fra* as well (Table 1). Similarly, no transheterozygous interaction between *slit* and *mid* was observed (Table 1). Therefore, we conclude that no transheterozygous interaction occurs between *mid* and *fra* or between *slit* and *mid*.

### Re-specification of NB row 2 into row 5 in *mid* mutants

We next sought to determine the molecular basis for the axon guidance defects in *mid* mutant embryos. Our results show that in *mid* mutant embryos some of the interneuronal pathways that normally project along the midline stall between PC and AC of the next segment and then get redirected across the midline or away towards the periphery, perpendicular to the midline (there are variations to this phenotype but the spectrum of such variations are all within this category). NBs are formed in waves (S1–S5) and in rows (1–7) under the control of neurogenic and proneural genes. Previous studies have shown that many of the segmentation genes, especially segment polarity genes, are expressed row-wise in NE and NB cells. These genes play a crucial role in the row-wise specification of NB identity [reviewed in ref. 15].

**Figure 6:**
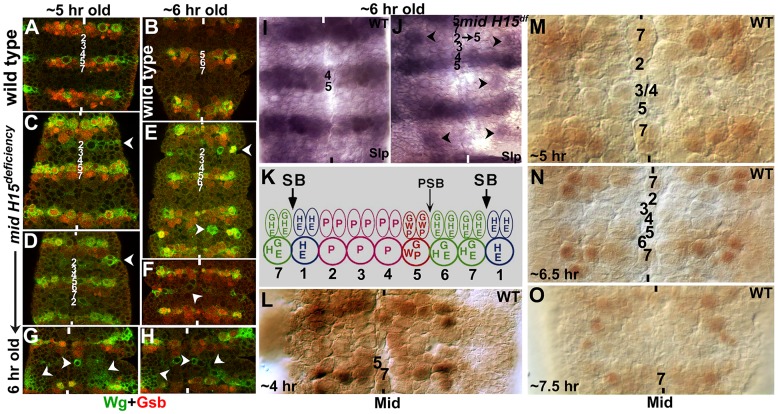
Wingless and Gooseberry are inappropriately expressed in row 2 cells in *mid* mutants. **Panel A–H:** Wild type (A, B) and mutant embryos (C–H) were double-stained with antibodies against Wg (Green) and Gsb (Red). Anterior end is up, midline is marked by vertical lines. Rows of NBs are numbered in both 5 hours old and 6 hours old embryos. Arrow-head in mutant embryo panels indicates ectopic Wg (and less frequent ectopic Gsb) expression. Note that in some hemisegments, ectopic Wg expression is extensive (panels G and H). **Panels I and J:** Slp expression in wild type and mutant. Note the ectopic Slp in mutant embryos, indicating transformation of row 2 into row 5 NBs (arrowheads). **Panel K:** Expression of some of the major segment polarity genes in the neuroectoderm (NE) and neuroblasts (NB) is shown (saggital view). Two rows of NE cells correspond to one row of NBs (each NB is twice the size of a NE cell); thus, for example, Wg is expressed in two rows of NE cells whereas it is expressed only in one row of NB. Expression of Gsb in the most posterior row of NE cells is restricted to only the cells closest to the midline, from which NB7-1 is derived. **Panels L–O:** Expression of Mid in early embryonic neurogenesis in wild type. A strong Mid expression is seen in rows 7 and 1 and remains so throughout neurogenesis, whereas weak and varying expression of Mid can be observed in other rows of NBs and this expression pattern changes with time.

To determine if the row-wise cellular identity within the nerve cord is altered in *mid* mutants, which might underlie the inhibitory zone and the associated guidance phenotype, we sought to examine the expression of some of the segment polarity genes. First, we examined *mid* mutant embryos for the expression of Wingless (Wg or W in Fig. 6K) and Gooseberry (Gsb and G in Fig. 6K). In wild type, Wg is present in row 5 NBs and the corresponding NE cells (Fig. 6A, B, K, see also Bhat, 1998). In *mid* mutant embryos, row 5 NB or NE expression of Wg was not affected, however, we observed ectopic expression of Wg in row 2 NBs and the corresponding NE cells (Fig. 6C–H). This ectopic expression was often stronger in alternate segments (see Fig. 6C, D, E). We note that the extent of ectopic expression of Wg was variable from segment to segment. For example, we found hemisegments or segments in which large patches of cells in the region between row 5 and row 7 (of the preceding segment) expressing ectopic Wg (Fig. 6G and H), which can also explain some of the variations in the guidance defects. Nonetheless, these results indicate that cells in row 2 behave as if they were row 5 cells. That this mis-expression occurs during segmentation is also indicated by the cuticle defects seen in *mid* mutant embryos, with missing denticle belts in the corresponding region (see Text S1 and Fig. S1).

Consistent with the above interpretation of Wg results, Gsb expression was also mis-expressed in *mid* mutant embryos. In wild type, Gsb is expressed in rows 5, 6 and one NB in row 7 (NB7-1). In *mid* mutant embryos, while the normal expression of Gsb in rows 5, 6 and NB7-1 was not affected, we observed ectopic expression of Gsb in the same cells expressing ectopic Wg (Fig. 6E). However, unlike the ectopic Wg stripe, which was always present in the mutant embryos at detectable levels, the ectopic expression of Gsb in the stripe was often incomplete and at times undetectable. Occasionally, we observed strong ectopic Gsb expression corresponding to both row 5 and row 6 cells suggesting that in *mid* mutants in addition to row 2 cells changing into row 5 cells, some row 3 cells may change into row 6. Though infrequent, in such segments it appears there is a reiteration of row 5 and 6 (rows 1, 5, 6, 4, 5, 6, 7) to varying degrees within the nerve cord in *mid* mutant embryos.

We next stained the mutant embryos for the expression of Sloppy-paired (Slp). We decided to examine Slp expression since in wild type Slp is expressed in rows 4 and 5 [Fig. 6I; see also ref. 13] and a change in Slp expression in *mid* mutants would allow us to confirm the results from the Wg and Gsb staining. This would also help us determine if cells corresponding to row 4 have changed to some other row of cells. In *mid* mutant or deficiency embryos, we observed ectopic expression of Slp in cells corresponding to row 2 cells (possibly some cells from row 3)(Fig. 6J). However, the ectopic expression of Slp was stronger in those segments where ectopic Wg was also strongly expressed. Again, the ectopic Slp expression was incomplete compared to ectopic Wg. Nonetheless, these results show that multiple row 5-specific segmentation genes are expressed in row 2 cells in *mid* mutant embryonic CNS.

Our previous results have shown that Mid is strongly expressed in row 7 and row 1 cells as well as in corresponding midline cells [20]. Since the expression of key genes can change quickly from division to division in NBs, and is highly time-sensitive [16], [17], we re-examined wild type embryos with an antibody against Mid. As shown in Fig. 6L–O, we found that Mid is indeed expressed at low levels in a large number of NBs, including in rows 2, 3 and 4 (perhaps also in one NB in row 5). Except for the strong expression in row 7 and row 1, which remained unchanged during neurogenesis, the expression pattern of Mid in other NBs changed as neurogenesis proceeded (Fig. 6L–O).

### Longitudinal axon bundles stop at the boundary of re-specified rows in *mid* mutants

**Figure 7:**
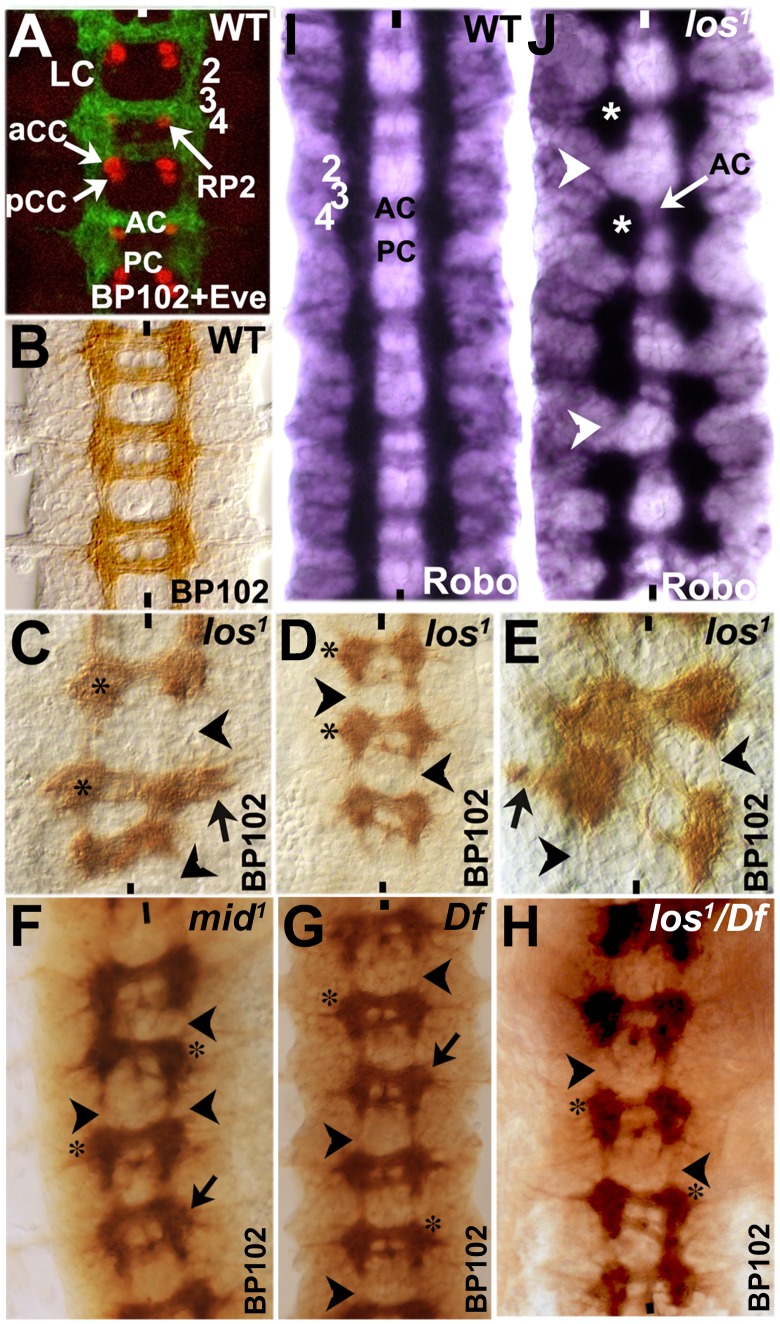
Longitudinal axon tracts stall just before the region of ectopic Wg and Gsb expression in *mid* mutant embryos. Wild type (panels A, B, I) and mutant (panels C–H, J) embryos were stained with BP102 and Eve (panel A), BP102 (panels B–H), and Robo (panels I and J). Anterior end is up, midline is marked by vertical lines. aCC/pCC and RP2 are Eve-positive neurons. The numbers 2, 3, and 4 indicates approximate location of NB rows 2, 3 and 4 formed earlier during neurogenesis. Neurons formed from a NB generally stay in the same location as the parent NB [see ref. 28]. For example, aCC/pCC pairs are formed from NB1-1 in row 1; RP2 is formed from NB4-2 in row 4. AC, anterior commissure; PC, posterior commissure; LC, longitudinal connectives; star indicates stalled blobs of axon tracts. Arrowhead indicates missing longitudinal axon tracts; black arrow indicates outward projection of longitudinal tracts and star indicates stalled axon tracts.

If we stain wild type Drosophila embryos with a monoclonal antibody BP102, we can clearly visualize commissural architecture with the longitudinal axon tracts (LC) and the anterior and posterior commissures (AC and PC; see Fig. 7A, green and Fig. 7B). Unlike the Fas II or other markers examined in the preceding sections, which are all directed against a small number of axon tracts, BP102 recognizes many more CNS axons and provides a more complete picture of axon tracts within the nerve cord. Therefore, we double stained embryos with BP102 and an antibody against Even-skipped (Eve) to determine the position of certain Eve-positive neurons (and therefore their parent NBs) in relation to the commissural architecture. Eve staining shows that an RP2 neuro, which is generated by NB4-2 (a row 4 NB), is located at the inner armpit of AC [Fig. 7A; see also ref. 28], affirming the position of row 4 NBs at the level of the posterior border of AC. While RP2 undergoes a complex migration within the nerve cord during development, ultimately it settles down in the same row where its parent NB is formed [28]. Similarly, the Eve-positive aCC/pCC neurons are located at the inner armpit of PC (Fig. 7A), which are generated by NB1-1, thus, fixing the location of row 1 to the posterior border of PC. Unlike the RP2, aCC/pCC neurons do not undergo much migration and stay in the same row 1 [28]. Thus, although NBs generate numerous progeny and there is both germ band retraction and condensation of the nerve cord, the relative position of commissures at a later point in neurogenesis to early NB rows remain more or less stable.

We next stained embryos from different alleles of *mid* (*los^1^, mid^1^, mid H15 df and los^1^/mid, H15 df*) with BP102 (Fig. 7C–H) to visualize the commissural architecture in mutant embryos (we did not double stain mutant embryos with BP102 and Eve in order to be able to flatten the nerve cord to fully visualize the commissural architecture and also to maximize the number of mutant embryos examined; the double staining shown in Fig. 7A was done to determine the relative position of AC and PC to rows of NBs). As shown in Fig. 7C–H, significant disruption of the commissural tracts was observed in all the mutant alleles of *mid*. We could clearly observe blobs of tracts or tracts projecting laterally (black arrow in 7C, D–G) at the level of AC, breaks in LC, as well as mis-projection of commissures between two adjacent neuromeres, creating a criss-cross phenotype (panel 7E; this criss-cross phenotype was observed in other alleles/combinations as well, data not shown). These tracts appear to encounter a non-permissive region for LC projection at row 2/3 (which lies just above AC). Furthermore, the posterior commissural (PC) tracts are reduced to only a few axon pathways in nearly all commissures, indicating a loss of axon tracts in PC. This may be a secondary effect of stalling of axon tracts in preceding neuromeres, thus, reducing the number of axon tracts that cross the midline through PC. The anterior commissural (AC) tracts were also affected but to a lesser degree. In general, in all *mid* alleles, more than 80% of the hemisegments had longitudinal tracts stalled at the AC level, resulting in breaks above AC. However, it appears that *los^1^* has slightly more severe overall CNS defects compared to other alleles or the deficiency and this appears to be the case in embryos transheterozygous for *los^1^/Df* as well. This may be consistent with the possibility that this specific allele has some gain of function effects given the molecular lesion in the gene in this allele [20]. Nevertheless, the defects were similar in all alleles.

Although Robo is present at very low levels in commissural tracts [Robo is down–regulated in commissures, see ref. 1], the Robo-staining pattern closely resembles that of BP102, minus the strong commissural staining of BP102 (Fig. 7I). With Robo staining of *mid* mutants, we could observe that the longitudinal axon tracts stall at the AC level in all *mid* mutants (see Fig. 4 also). This corresponds to row 3 NBs in wild type, which is just before row 2 (re-specified as row 5 in the mutant). It appears that, when longitudinal axon tracts encounter the re-specified row 5 cells in *mid* mutants, they stop and simply congregate at this position, forming a blob (Fig. 7J, see also C–H, indicated by star). This is also evident by the breaks in the continuity of longitudinal tracts (Fig. 7J, arrowhead). These defects are consistent with the presence of a region or a barrier above AC that is not permissive to longitudinal axon projection. These results argue that loss of function for *mid* alters the identity of rows of NE and NB cells. By the time neurons begin to project their growth cones, this change of row identity creates a zone which is either non-permissive or lacks signals for growth cones to continue in their usual path. Thus, these growth cones either collapse on to themselves or project laterally outward, or in some segments/hemisegments cross the midline in this region (see Fig. 2 also).

## Discussion

Guiding axon growth cones towards their synaptic targets is one of the most fundamental processes during neurogenesis. Axon growth cones navigate through different regions by responding to cues from the environment to ultimately find their synaptic targets. While the two major signaling pathways, Slit-Robo and Net-Fra, provide a larger architecture for axon guidance within the nerve cord, local environment is expected to influence axon guidance as well. However, this particular aspect has not been examined in detail. A given local environment will be determined by the identity of the neuroectoderm, neurons, ectoderm and perhaps by the identity of the mesoderm as well. Thus, broad changes in local environment in which axon growth cones have to navigate is likely alter the route or guidance of these axons.

It is well established that segment polarity genes determine the broad identity of cells within the nerve cord just as they do later during development to determine the segmental identity within the epidermis [reviewed in ref. 15]. Segmentation genes, specifically the segment polarity genes such as Wg, Ptc, Hh, En are expressed in rows of NBs to define specific and row-wise NB identities. That these segmentation genes also play a role in axon guidance is indicated by the fact that mutations in many segmentation genes alter axon guidance [18]. However, given that these mutations also alter NB identity, the effect of mis-specification of neuronal identity versus broad changes in the environment in which axons navigate, on axon guidance has been experimentally difficult to separate.

Our work described in this paper, however, attempts to separate the role of identity versus environment and reveal the significance of local environmental niche to normal axon guidance. Our results show that in *mid* mutants, there is an ectopic expression of segment polarity genes such as Wg, Gsb, Slp (and perhaps many more) in row 2 cells within the developing nerve cord, thus, re-specifying this row of cells into more like row 5 cells (and a second PSB). This re-specification appears to ultimately create a zone or a barrier that prevents axon growth cones from progressing further in their normal route. Instead, such growth cones either stall or project peripherally or across the midline but along this zone of non-permissive barrier. The highly specific nature of the phenotype(s) in response to a specific change in the environment in *mid* mutants presents a classic example of the specificity of the environment to axon guidance. Our results also show that the identity of some of the pioneering neurons, whose axon projections are misrouted, is not affected by loss of function for *mid*.

It would have been ideal if we were able to identify a single molecule that makes this re-specified row of cells non-permissive to longitudinal tracts extension in their usual path. We do not know if such a molecule exists, or the mechanism that created the barrier. But the barrier is unlikely due to the ectopic expression of genes such as Wg, instead it must be due to the change in the row-identity, activating a distinct genetic program that does not permit axonal extension in their normal path. Ectopic expression of Wg, or Slp or Gsb simply reflects this change. We also point out that this re-specified row 5 cells may not have all the features/genetic programs of a bona fide row 5 cells and more likely have a mixed identity. This is suggested by the fact that Slp and Gsb or even Wg expression in the re-specified row is not exactly like in a bona fide row 5. Similarly, we do not know at what point in development this zone or barrier is put in place, but it indeed originates with the altered row identity, and certainly becomes active by the time of pathfinding. This barrier might be due to signals from other neurons generated by the transformed NB row, or the transformed neuroectoderm/ectoderm. We further point out that a clonal analysis experiment would have been desirable to show that a broad identity-change is necessary for the observed guidance defects. However, *mid* is expressed extensively in the germline, both in the soma and the germ cells, and there is a requirement for Mid in these cells. Ultimately, it may require isolation of a temperature sensitive allele in *mid* to address this question in an unequivocal way.

### Mid and axon guidance genes

Our results show that Mid does not regulate the expression of *slit*, *robo* or *fra* genes in the CNS. Consistent with this, the axon guidance defects in *mid* are distinct from the defects in *slit* or *robo* mutants. We confirm this by several different ways: immunostaining, RNA whole mount in situ, Western analysis, qPCR and genetic interaction studies. A previous study has suggested that Mid regulates *sli, robo and fra* [27]. They based their conclusion on finding a strong transheterozygous genetic interaction between *mid* and *sli*, and *mid* and *fra*, detected using BP102 staining of embryos that are transheterozygous for *mid^1^* and *sli^GA20^* and *mid^1^* and *fra^3^*. Furthermore, they reported that levels of *fra* mRNA and Fra and Robo proteins in *mid* mutant embryos were down regulated, and that this can be completely rescued by expressing *mid* using elev-GAL4 driver [27]. They also reported that ectopic expression of *mid* in salivary glands induces expression of *robo* and *slit*. We have not found most of these effects reported by Liu et al [27]. For example, we failed to observe any genetic transheterozygous interactions between *mid* and *sli* mutants (Table 1). Transheterozygous interactions are rare given the negative evolutionary impact of such interactions to survival, but when observed, it is usually with mutations in receptor-ligand pairs, or with gain of function/neomorphic situations). We used stronger allelic combinations than the ones used by Liu et al [27] with *mid* and *sli*. For *mid*, we used not only *mid^1^*, but also a deficiency that removes both *mid* and its sister gene H15, as well as *los^1^*. For *slit*, we used *sli^2^*, which is the strongest loss of function allele and genetically behaves as a null. Furthermore, we also failed to observe any such transheterozygous interactions between *mid* and *fra* (Table 1).

Secondly, we found that while the ectopic expression of *mid* in salivary glands induced *robo* expression as was reported by Liu et al [27], no such induction was observed with *slit* (Text S1 and Fig. S2). One should also consider the fact that Mid, Robo (and Slit) have mostly non-overlapping domains of expression in the CNS, therefore, the direct regulation of *robo* by Mid in the salivary gland has little relevance in the CNS or CNS development mediated by Robo or Mid. Our results indeed bears this out. Not only the axon guidance defects are different between *mid* and *slit* or *robo*, the transcription of *robo*, *slit* or *fra* are also unaffected in *mid* mutants (Figs. 3–5, Table 1). There was some reduction in the levels of Slit in *los^1^* allele in the midline in the PC region. But, the molecular lesion in *los^1^* is complex and might have some allele-specific gain of function effects that alters cellular identity or function of the corresponding midline glia to mediate reduction in the Slit level in this region. Since no such reduction in the levels are seen in other *mid* alleles and more importantly the transcription of *slit* is unaffected in the deficiency that removes *mid* (and *H15*), we think that the slight reduction in the levels of Slit in *los^1^* is allele-specific.

In the case of *robo*, the promoter has three TBEs. With three sites, Mid is more likely to be able to induce *robo* in an ectopic site. However, within the CNS, we did not find any significant loss of Robo expression in *mid* mutant embryos by immunohistochemistry (either in *los^1^* or *mid H15* deficiency embryos) or by Western analysis (Fig. 4) or *robo* transcription by qPCR (Fig. 5). A slight reduction in the levels of Robo seen in Westerns is likely due to a secondary effect of loss of tracts and perhaps loss of some of the Robo-expressing neurons perhaps due to identity changes [19]. The reason for the significant reduction in the expression of Robo in *mid* observed by Liu et al [27] is not clear. We think that this may be due to some technical reasons such as variability from embryo to embryo to fixing and staining. Because of this possibility, we follow a simple rule: in this case, we focused on *mid* mutant embryos that had strong guidance defects to determine if such mutant embryos also had a strong or weak expression of Robo and Slit. We found that embryos with strong guidance defects also had strong Robo (or Slit) expression. Thus, we avoided selecting sub-stained mutant embryos and comparing them to optimally stained wild type embryos. Finally, Liu et al [27] reported that there is overlapping expression of Mid and Slit in a small number of cells located laterally within the nerve cord. It is possible that Mid regulates *slit* expression in these cells, however, the contribution of Slit or such a regulation of *slit* to the overall axon guidance mediated by Slit is not clear and likely very minimal, if there is any. We have also not examined if *mid* affects *netrin* gene expression.

### Axon guidance defects in *mid*: Transformation of row 2 to row 5 cells blocks growth cone projection along the nerve cord

Our work shows that in *mid* mutants, the majority of axon growth cones of the longitudinal tracts stall and club together at the level of AC, creating a blob of axons, thus leading to interruptions between neuromeres. Interestingly, some of the tracts project outward towards the periphery or inward across the midline (see Table 1). This outward projection route is quite revealing: the projection path is mostly perpendicular to the midline and just below the transformed row of cells. The transformed row of cells corresponds to the region right above the AC or where the tracts stall (Figs. 6 and 7). The most consistent change is seen with row 2 cells, changing into row 5 cells. How does these changes relate to wild type? In wild type, row 5 cells normally separate one neuromere from the next and also define PSB. Thus, the change from row 2 to row 5 in *mid* must be creating an environment that either lacks the necessary permissive/attractive cues or possess cues that are inhibitory to the projection of these axon tracts, causing the tracts to stall.

For example, in wild type row 5 cells are located between pCC and vMP2, the two axons that pioneer the Fas II-positive medial tract. The growth cone from vMP2 in wild type only marginally encounters row 5 cells but does not necessarily traverse row 5. This is due to the fact that vMP2 is located in row 5 and the growth cone from a vMP2 stops at row 5 region and fasciculates with the vMP2 of the next hemisegment. However, it does encounter row 2 cells midway through the projection path. In *mid* mutants, since row 2 cells change to row 5, creating a region that vMP2 growth cone is perhaps normally programmed to stop. For the proper guidance of medial tract, normal projection of vMP2 and pCC is necessary and loss of either of the two pioneer neurons causes aberrant medial tract guidance [9], [10]. Therefore, it seems likely that vMP2 stalls and the pCC projection, along with several other follower projections, also stalls; or some of the tracts project across the midline or away towards the periphery. In fact, these abnormal projection patterns, especially towards the periphery appear to be guided by the newly created barrier. This situation is also the same for MP1 or dMP2.

What is the mechanism within the re-specified row of cells that eventually mediates the block for axon projection? The re-specified rows of cells would have a whole set of new (row 5-specific) genetic programs that may simply not conducive to longitudinal tracts. Additionally, the role of Ephrin pathway in axon guidance may be relevant here. The Drosophila Ephrin (Eph), which is a transmembrane protein, is shown to prevent interneuronal axons from exiting the Drosophila embryonic CNS [29]; some of the interneuronal pathways in *mid* mutant exit the nerve cord (Fig. 2G). Ephrin/Eph signaling is via cell-to-cell contact and depends on the clustering of Eph receptors and their ligands [30]–[33]. This multimerization activates the kinase activity of the receptor and leads to the phosphorylation of the receptor within the cytoplasm-exposed tail region and the binding of downstream effectors [34]. This triggers the depolymerization of actin in growth cones, modifying the Integrin-based cell adhesion [29], [35]. The CNS-exiting phenotype of interneuronal pathways in *mid* mutants suggests a possible de-regulation of the Eph-pathway. But, it may also be that changes in Eph or similar cell-adhesion mechanism mediate the formation of the barrier and exiting of some of the interneuronal pathways from the CNS.

Our previous results show that Mid acts as a transcriptional repressor of *gsb-n* [20]. However, in *mid* mutants the transformation of row 2 into row 5 also activates Gsb expression (Fig. 6). The ectopic activation of Gsb in these cells in *mid*, however, is not a direct de-repression of *gsb*, but an indirect consequence of the transformation of cell identity from row 2 (a Gsb-negative row of cells) to row 5, a Gsb-positive row. Finally, our results provide clear evidence that segmentation genes can regulate axon guidance via broadly defining cellular identity, creating a permissive and non-permissive boundaries or niche. We also emphasize that extrapolating expression relationships to functional relevance from induction in ectopic sites, in vitro and tissue culture experiments, bioinformatics or other similar in vitro studies carry inherent risks and should be done with caution.

## Materials and Methods

### Fly strains, genetics

*mid* mutant alleles used were *mid^1^*, *mid^2^* and *los^1^*. We also used a deficiency that removes both *mid* and *H15* genes (*mid H15^df^*; BL# 7498: breakpoints: 25D5-25E6). The other lines used were: *sli^2^, robo^4^, robo-deficiency* [Df (2R) BSC787, breakpoints: 58F4-59B1; BL#27359], *UAS*-*mid*, sim-GAL4, sgs-3-GAL4 (to induce *mid* in the salivary gland), ac-GAL4, *UAS-mCD8-GFP*, RN2-GAL4 (eve-GAL4) and *UAS-tau-lacZ*. For wild type, we used Oregon R flies. All the mutant lines were balanced using GFP-bearing balancer chromosomes to facilitate identification of the mutant genotype.

### *mid* induction in the salivary gland

Transgenic *UAS-mid* fly lines containing one or two copies of the *UAS-mid* were previously generated in the lab [20]. The transgenic flies were crossed to sim-GAL4. Embryo collection was done overnight at 28°C. The embryos were fixed, divided into three portions and stained separately with antibodies against Mid, Robo and Slit.

### Examining the axon tract from vMP2, dMP2 in mid mutant embryos

ac-GAL4 driver (BL#: 8715) and the *UAS-mCD8-GFP* (BL# 41803) were introduced into the *mid H15* deficiency background and the embryos were stained for GFP and Odd-skipped. ac-GAL4 drives the *UAS-mCD8-GFP* in vMP2 and dMP2 and their axons. Odd-skipped is expressed in dMP2 and MP1.

### Examining the axon tract from pCC, aCC and RP2 neurons

RN2-GAL4 (portion of the *eve* promoter that drives expression in aCC/pCC and RP2 neurons) and *UAS-tau-lacZ* were introduced into *mid H15* deficiency background and the embryos were stained for LacZ.

### Examining the axon tract from MP1

sim-GAL4 and *UAS-tau-GFP introduced to mid H15* deficiency background and stained for GFP and Eve (Eve to identify the mutant). Tau and MCD8 targets GFP to axon tracts.

### Immunohistochemistry and whole mount RNA in situ

The embryo collection, fixation and immunostaining were performed according to the standard procedures. The following antibodies were used: anti-Sli C (1∶20, DHSB), anti-Robo (1∶5, DHSB), anti-Mid [1∶50, generated in the lab, see ref. 20], anti- Fas II (1∶5, DHSB), 22C10 (1∶1, DHSB), anti-Wg (1∶5), anti-Gsb (1∶3), anti-Slp (1∶400), anti-GFP (1∶300), anti-Odd (1∶500), anti-Eve (1∶2000), anti-Lac Z (1∶500). For color visualization, either AP-conjugated or HRP-conjugated secondary antibodies were used. For double staining, secondary antibodies conjugated with AlexaFluor488 and AlexaFluor635 were used. Whole-mount RNA in situ hybridization for *sli* expression was done following the standard procedure using a digoxigenin-labeled *slit* probe, synthesized by PCR.

### Cuticle preparation

Cuticle preparation was done as per standard procedure by fixing embryos and dissolving organic embryonic material on slides using Hoyer's solution at 65°C for 24 hours.

### Western analysis

For western blot analysis, 30 embryos were collected (homozygous mutant embryos were identified by the lack of GFP expression under microscope), homogenized in 37.5 µL lysis buffer (0.15 M NaCl, 0.02 M Tris pH,7.5, 0.001M EDTA, 0.001 M MgCl2, 1% Triton-X-100 and PIC) and kept on ice for 10 minutes. The lysed protein is centrifuged for 5 minutes at 13,000 rpm, the supernatant is collected and diluted with 12.5 µL 4× Laemelli sample buffer. The protein sample is boiled in water for 10 minutes and kept in 4°C for 10 minutes. Equal amount of lysed protein 20 µL (15 embryos per lane) was loaded on to a 4–12% SDS-PAGE gel. The separated proteins were transferred to a Nitrocellulose membrane. The efficiency of transfer was determined by Ponceau S staining. The membrane is blocked in 5% milk for 2 hours at room temperature, and incubated with primary antibodies (anti-Slit N 1∶50000 or anti-Robo 1∶40) overnight at 4°C and washed with PBST (PBS+0.02% Tween 20). The Nitrocellulose membrane was then incubated with HRP-conjugated secondary antibody (anti-Rabbit 1∶20000 or anti-mouse 1∶20000) for 2 hours at room temperature and washed with the washing buffer. Proteins were detected by the chemi-luminiscent ECL reaction method (Thermo Scientific). The autoradiographs were scanned and intensities of bands were analyzed using the software AlphaEaseFC. Anti-Tubulin antibody (Abcam, 1∶4000) was used for determining loading control.

### Real-time Polymerase chain reaction (PCR)

Embryos from Oregon R (wild type) and *mid H15* deficiency lines were collected and aged for 12–14 hours. They were dechorionated in 50% bleach and washed with water. Approximately 150 embryos were selected under microscope for each sample. Total RNA isolation from these embryos were performed using the RNaqueous Kit (Ambion). The isolated RNA was DNase treated and quantified using Nanodrop Spectrophotometer (Nanodrop Technologies) and qualified by analysis on RNA Nanochip using Agilent 2100 Bioanalyzer (Agilent Technologies). Synthesis of cDNA was performed with 1 µg of total RNA in a 20 µL reaction using the Taqman Reverse Transcription Reagents Kit (ABI). Reaction conditions were as follows: 25°C, 10 minutes, 48°C, 30 minutes and 95°C, 5 minutes. Primers for real-time PCR were designed and made by the Molecular Genomic Core facility at UTMB. Real-time PCR were done using 1.0 µL of cDNA in a total volume of 20 µL using the Faststart Universal SYBR green Master Mix (Roche, #04913850001). RpL32 was used as endogenous control. All PCR assays were performed in the ABI Prism 7500 Sequence Detection System and the conditions were as follows: 50°C, 2 min, 95°C, 10 min, 40 cycles of 95°C, 15 sec and 60°C, 1 min. Primers used: *slit*: Forward: 5′-GCGTTATGCCCGGTTCC-3′, Reverse: 5′ TCCACAACGTGCCGCTC-3′); *robo*: Forward: 5-CAGCATTAGTCTTCGTTGGGC-3, Reverse: 5-AATCCAACCAGTTTGCAGATTC-3); *fra*: Forward: 5-AGACCCCAGAGCATCCTTATG -3, Reverse: 5-TCTTTAGAGGATGGCCACGC-3. The qRT-PCR was done on three seperate embryo collections for each genotype and in triplicates for each collection.

## Supporting Information

Figure S1 Cuticle defects in *mid* mutants reflect ectopic expression of Wg in NE cells. Cuticle preparation from wild type (A) and mutant (C–E) embryos are shown. A1, abdominal segment 1, A2, abdominal segment 2. Cuticle denticle belts defects in *mid* includes missing rows (arrowhead), particularly the row 2 belt (panel D), to complete absence in a half-segment or from the midline (arrows, panel E). In panel B, saggital view of epidermal cells alternating the denticles and naked region and the expression pattern of segment polarity genes is shown (∼15 hour old embryo). Numbers 1–6 represent the type of denticles secreted by these epidermal cells in rows. The first row denticles (Type 1) are small and point anteriorly and are secreted by Engrailed (En) and Hedgehog (Hh) expressing cells. The second row (Type 2) denticles are longer and point posteriorly. The 3rd row (Type 3) are very similar to (Type 2), whereas in row 4 or (Type 4), the denticles are small and point anteriorly. The fifth row is large and thick and point posteriorly, whereas the sixth, are very small and also point posteriorly. The rest of the segment consists of naked cuticle, which is primarily defined by Wg and Gsb expression. P, Ptc; G, Gsb; H, Hh; and E, En; SB, segmental boundary; PSB, parasegmental boundary.
(TIFF)

Figure S2 Induction of Mid in the salivary gland induces Robo but not Slit induction. Mid was induced from a *UAS-mid* transgene using the sim-GAL4 [salivary gland (panel A) and midline specific] and sgs3-GAL4 (salivary gland specific) drivers. Both drivers induced expression of Mid in salivary glands (panels B and E), and induction of Robo in salivary gland (panels D and G) but not Slit (panels C and F). Arrows indicate salivary glands.
(TIFF)

Text S1 Epidermal cell identity and ectopic induction of Robo but not Slit by Mid. The first part of the text describes our results that in *mid* mutants, ectopic Wingless expression affects epidermal cell identity as well, with reiteration of naked cuticle corresponding to row 2 NBs. In the second part of the text, we show that ectopic expression of *mid* in salivary gland induces expression of Robo, but not Slit.
(PDF)
