# Supplementary figures and images for: The Midline Protein Regulates Axon Guidance by Blocking the Reiteration of Neuroblast Rows within the Drosophila Ventral Nerve Cord
Source: PLoS Genet. 2013 Dec 26;9(12):e1004050. doi: 10.1371/journal.pgen.1004050 (PMC3873230; doi:10.1371/journal.pgen.1004050)

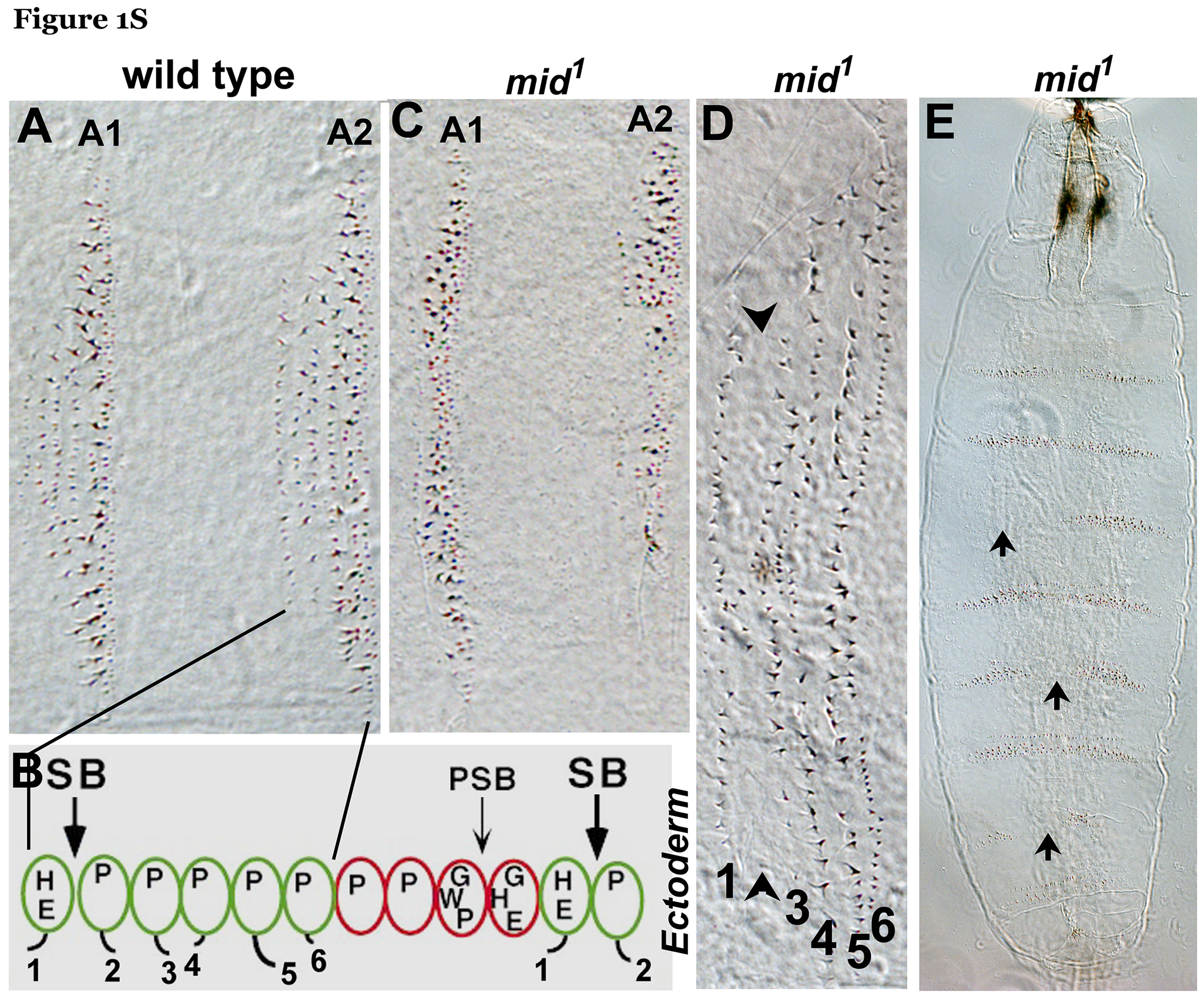

Supplement: Figure S1 — Cuticle defects in mid mutants reflect ectopic expression of Wg in NE cells. Cuticle preparation from wild type (A) and mutant (C–E) embryos are shown. A1, abdominal segment 1, A2, abdominal segment 2. Cuticle denticle belts defects in mid includes missing rows (arrowhead), particularly the row 2 belt (panel D), to complete absence in a half-segment or from the midline (arrows, panel E). In panel B, saggital view of epidermal cells alternating the denticles and naked region and the expression pattern of segment polarity genes is shown (∼15 hour old embryo). Numbers 1–6 represent the type of denticles secreted by these epidermal cells in rows. The first row denticles (Type 1) are small and point anteriorly and are secreted by Engrailed (En) and Hedgehog (Hh) expressing cells. The second row (Type 2) denticles are longer and point posteriorly. The 3rd row (Type 3) are very similar to (Type 2), whereas in row 4 or (Type 4), the denticles are small and point anteriorly. The fifth row is large and thick and point posteriorly, whereas the sixth, are very small and also point posteriorly. The rest of the segment consists of naked cuticle, which is primarily defined by Wg and Gsb expression. P, Ptc; G, Gsb; H, Hh; and E, En; SB, segmental boundary; PSB, parasegmental boundary. (TIFF) [file pgen.1004050.s001.tiff]

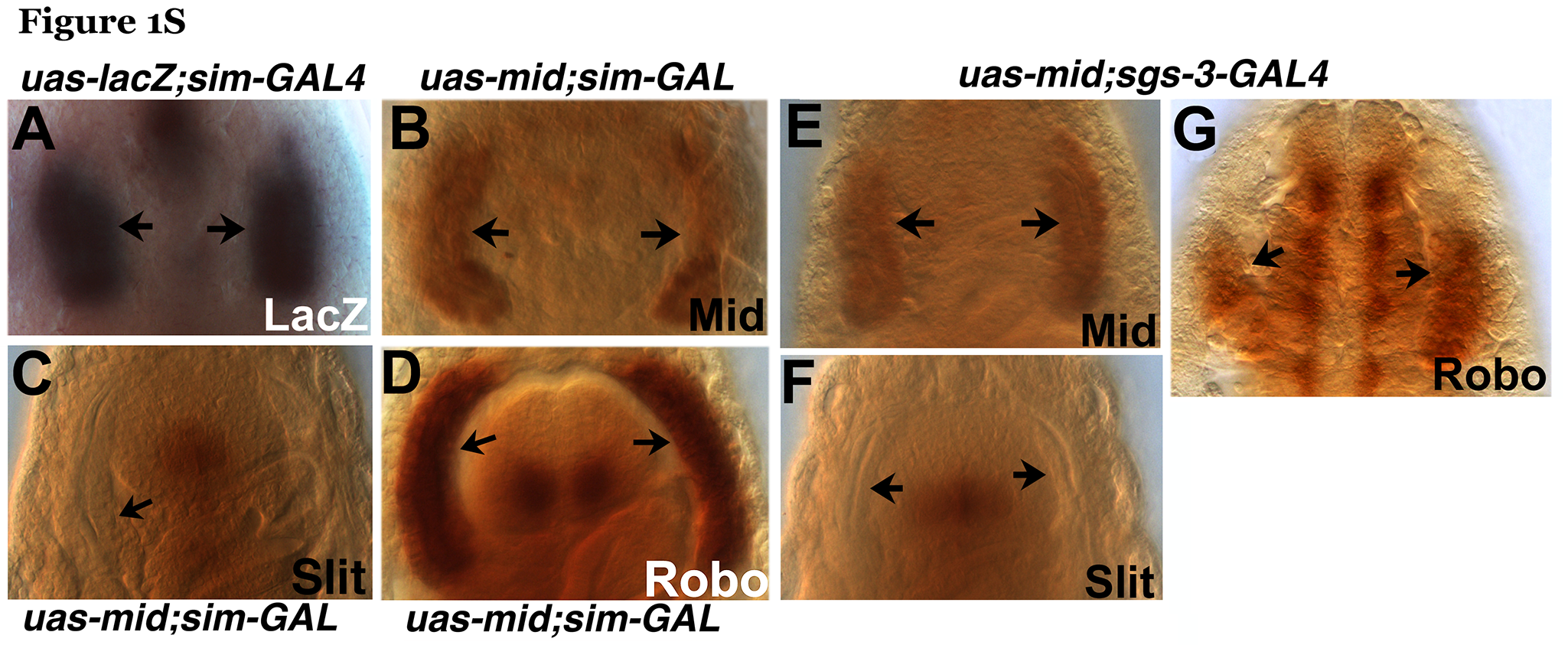

Supplement: Figure S2 — Induction of Mid in the salivary gland induces Robo but not Slit induction. Mid was induced from a UAS-mid transgene using the sim-GAL4 [salivary gland (panel A) and midline specific] and sgs3-GAL4 (salivary gland specific) drivers. Both drivers induced expression of Mid in salivary glands (panels B and E), and induction of Robo in salivary gland (panels D and G) but not Slit (panels C and F). Arrows indicate salivary glands. (TIFF) [file pgen.1004050.s002.tiff]
