# Supplementary material for: The Midline Protein Regulates Axon Guidance by Blocking the Reiteration of Neuroblast Rows within the Drosophila Ventral Nerve Cord
Source: PLoS Genet. 2013 Dec 26;9(12):e1004050. doi: 10.1371/journal.pgen.1004050 (PMC3873230; doi:10.1371/journal.pgen.1004050)
Supplement: Text S1 — Epidermal cell identity and ectopic induction of Robo but not Slit by Mid. The first part of the text describes our results that in mid mutants, ectopic Wingless expression affects epidermal cell identity as well, with reiteration of naked cuticle corresponding to row 2 NBs. In the second part of the text, we show that ectopic expression of mid in salivary gland induces expression of Robo, but not Slit. (PDF) [file pgen.1004050.s003.pdf]

Supporting Information

Ectopic Wingless expression in *mid* mutants affects epidermal cell identity as well

Cell commitment into neural lineages occurs prior to ectodermal commitment [reviewed in ref.15]. The ectoderm in wild type is divided into a denticle belts region and a naked region (Fig. S1A, B). There are 6 rows of denticle belts with each belt comprised of a different type of denticles (see Fig. S1B for graphic representation). The denticle region is primarily defined by the expression of Hedgehog (Hh), Engrailed (En) and Patched (Ptc), whereas the naked region is defined by Wg and Gsb (Fig. S1B). Since ectopic Wg, Gsb and Slp in *mid* mutant embryos is observed in the precursor neuroectodermal cells and NBs, we sought to determine if this ectopic expression, especially of Wg, results in an altered cuticle pattern. This is because Wg is involved in defining naked cuticle and in loss of function for *wg*, the naked cuticle is replaced by denticle belts [reviewed in ref.15]. Conversely, gain of function for Wg causes naked cuticle. We reasoned that if the ectopic Wg expression also occurs in the ectoderm, it should give rise to naked cuticle in the region of ectopic Wg stripe in *mid* mutants.

The original mutations in the *mid* gene were identified as causing the loss of cuticle at the midline region (thus, the name *midline*)[25]. We performed a more detailed analysis of the cuticle pattern in *mid* mutants and found that, in addition to loss of cuticle in the midline region (Fig. S1E, mid-arrow), there were additional defects a such as missing denticle belts from several rows (Fig. S1C, D), which are consistent with ectopic Wg expression. Occasionally, loss of denticles in *mid* embryos could be observed in entire half segments (Fig. S1E on the upper left). Such loss of denticles from entire half-segments is also consistent with the occasional ectopic expression of Wg in most of the NE cells in a half-segment (see Fig. 6G, for example).

Mid can ectopically induce Robo but not Slit expression in salivary glands

Liu et al had previously reported that Mid can ectopically induce Slit and Robo in salivary gland [27]. We had previously generated *UAS-mid* transgenic lines [20] and we used these transgenic lines to induce *mid* with the driver *single-minded (sim)-GAL4*. We selected sim-GAL4 as our driver since it is strongly active in the salivary gland (Fig, S2A) in addition to the midline. *mid* was induced in all cells of the salivary gland (Fig. S2B). We next examined the expression Slit in the salivary gland in *UAS-mid; sim-GAL4* embryos using Slit antibody. However, in contrast to a previous report [27], we did not observe expression of Slit in salivary glands, even when UAS-*mid* was induced at 28 ^{0}C (Fig. S2C). Moreover, we also did not observe over-expression of Slit in the midline (data not shown). Furthermore, we could not induce Slit expression with *UAS-mid* transgene in salivary glands using the salivary gland specific driver sgs-3-GAL4 (Fig. S2F), even though Mid was expressed at high levels in salivary glands (Fig. S2E).

We next examined the expression of Robo in *UAS-mid; sim-GAL4* and *UAS-mid;* sgs-3-GAL4 embryos. As was reported previously [27], expression of Robo was observed in salivary glands (Fig. S2D and G). The expression of Robo was also induced in the midline with *sim-GAL4*, although this expression was relatively low and not in all midline cells (data not shown) although all midline cells expressed Mid. These results indicate that Mid is capable of ectopically activating *robo*, but not *slit*. Mid and other T-box proteins bind TBE to activate transcription [19], although promoter analysis of the actual target genes reveals that TBEs are highly variable in sequence, number and distribution. While the promoter region of *robo* has 3 such TBE sites, *slit* has only one, which appears to not be enough to activate *slit* transcription in vivo.
